# Activation of Kupffer Cells Is Associated with a Specific Dysbiosis Induced by Fructose or High Fat Diet in Mice

**DOI:** 10.1371/journal.pone.0146177

**Published:** 2016-01-05

**Authors:** Gladys Ferrere, Anne Leroux, Laura Wrzosek, Virginie Puchois, Françoise Gaudin, Dragos Ciocan, Marie-Laure Renoud, Sylvie Naveau, Gabriel Perlemuter, Anne-Marie Cassard

**Affiliations:** 1 UMR996 - Inflammation, Chemokines and Immunopathology -, Inserm, Univ Paris-Sud, Université Paris-Saclay, 92140, Clamart, France; 2 Univ Paris-Sud, DHU Hepatinov, Labex LERMIT, Clamart, 92140, France; 3 IPSIT, IFR141, Clamart, 92140, France; 4 AP-HP, Hôpital Antoine Béclère, Service d’hépato-gastroentérologie, Clamart, 92140, France; INRA, FRANCE

## Abstract

The increase consumption of fructose in diet is associated with liver inflammation. As a specific fructan substrate, fructose may modify the gut microbiota which is involved in obesity-induced liver disease. Here, we aimed to assess whether fructose-induced liver damage was associated with a specific dysbiosis, especially in mice fed a high fat diet (HFD). To this end, four groups of mice were fed with normal and HFD added or not with fructose. Body weight and glucose sensitivity, liver inflammation, dysbiosis and the phenotype of Kupffer cells were determined after 16 weeks of diet. Food intake was increased in the two groups of mice fed with the HFD. Mice fed with HFD and fructose showed a higher infiltration of lymphocytes into the liver and a lower inflammatory profile of Kupffer cells than mice fed with the HFD without fructose. The dysbiosis associated with diets showed that fructose specifically prevented the decrease of Mouse intestinal bacteria in HFD fed mice and increased *Erysipelotrichi* in mice fed with fructose, independently of the amount of fat. In conclusion, fructose, used as a sweetener, induced a dysbiosis which is different in presence of fat in the diet. Consequently, the activation of Kupffer cells involved in mice model of HFD-induced liver inflammation was not observed in an HFD/fructose combined diet. These data highlight that the complexity of diet composition could highly impact the development of liver lesions during obesity. Specific dysbiosis associated with the diet could explain that the progressions of liver damage are different.

## Introduction

Non alcoholic fatty liver disease (NAFLD) is associated with obesity, insulin resistance, diabetes, hypertriglyceridemia and arterial hypertension in the metabolic syndrome [1]. With the increasing incidence of obesity, NAFLD becomes probably the most common cause of chronic liver disease in Western countries. NAFLD can progress from steatosis to steatohepatitis (NASH, non-alcoholic steatohepatitis), fibrosis, cirrhosis and hepatocellular carcinoma [2]. NASH is characterized by steatosis associated with liver inflammation and liver immune dysregulation. Recruitment of inflammatory cells into the liver and their subsequent activation are the key steps in the progression of liver disease. NAFLD is associated with alterated hepatic lymphocyte subsets [3] including reduced numbers of hepatic Natural Killer T (NKT), lymphocytes and T regulatory lymphocytes. Moreover, resident macrophages of the liver, Kupffer cells (KCs) play a key role in the onset of NASH. We have recently showed that KCs of obese mice are loaded with lipid droplets named “fat-laden” KCs. This accumulation of lipids orients KCs towards a pro-inflammatory phenotype and participate to the development of an abnormal immune response in the liver [4].

Modifications of diet habits in western countries show an increase of fructose intake [5]. Indeed, daily fructose intake increased from 15g before 1900 until an estimated consumption of 73g in american adolescents in 1994. This increase is the reflect not only of a higher fruit juice intake but also of a more commonly substitution of sucrose by high fructose corn syrup in soda. Then, the percentage of fructose in the diet was increased from 4% until 12% of total calories [6]. We therefore aimed to study the involvement of fructose on the development of inflammatory liver lesions in mice fed a high fat diet. Fructose could be a specific substract for gut bacteria, we assessed dysbiosis caused by fructose enrichment specifically associated with high fat diet consumption.

## Materials and Methods

### Animal trials and diets

Mice were treated in accordance with the Guide for the Care and Use of Laboratory Animals (National Research Council, 1996).

The evaluation of installation was performed by the departement of veterinarian service « des Hauts-de-Seine » and the agreement number is C92-02-301. The relevant Institutional Animal care comittee that approved this work is the “Consortium des Animalerie Paris Sud » (CAPSud) registered with the comittee “Comité d’Ethique en Expérimentation Animale” under the number 26 (CEEA26). This work is anterior to the obligative ethic comittee but the technics, diet and treatement used in this work are currently used and were validated by the ethic comittee with the number 2014_009.C57BL/6J mice were purchased (Janvier, Le Genest, France) and maintained under a 12h light/dark schedule, with food and water *ad libitum* and treated in accordance with the Guide for the Care and Use of Laboratory Animals (National Research Council, 1996). Mice were fed either a normal diet (ND) or a high-fat diet (HFD) in which the energy content of fat was either 12% or 60% respectively. The HFD diet was performed with lard (34%) and contained saturated fatty acid (8.3%), mono-insaturated fatty acid (4.5%) and poly-insaturated fatty acid (5.8%), (Ssniff, Soest, Germany). Animals had free access to either water or water containing 20% fructose (w:v). Eight mice per group were fed the diets for 16 weeks. The caloric intake was measured by the diet consumption and the water containing fructose consumption. The energy content of normal diet (genestil 1314,Royaucourt, France) was 2988 kCal/kg and the energy content of the high fat diet (Ssniff D12492) was 5736 kCal/kg. The energy content of fructose (21%) was 84 kCal/L.

### Glucose tolerance

Oral glucose tolerance test (OGTT) were performed as follow: a glucose load (2 g/kg) was given by gavage after 6h of fasting and blood samples were taken from the tail vein at 0, 15, 30, 60, 90 and 120 min after the gavage. Serum glucose concentrations were determined by the Accu-Chek^®^ Performa (Roche, Risch, Switzerland) and the area under the glucose–time curve was calculated.

### Tissues and samples

Mice were anesthetised with ketamine (Imalgène, 200mg/kg) and Rompun (Xylasine, 20mg/kg) diluted in NaCl 0.9% and blood samples were collected in EDTA coated tubes. Then, the livers were perfused inversely to the normal flux with PBS/EDTA (5mM). After removing blood, livers were excised, weighed and either fixed in buffered formaldehyde or snap-frozen in liquid nitrogen for triglycerides (TGs) and RNA extractions. All samples were stored at -80°C until use. The serum was used for alanine aminotransferase (ALT), aspartate aminostransferase (AST) (Olympus AU400, Sydney, Australia), triglycerides, free fatty acids (FFA), HDL-cholesterol and Alkaline phosphatase (ALP) quantification by using a AU400 chemistry analyzer. One sample of blood was extemporanelly used to perform monocytes labelling.

### Isolation and labelling of NPC, KCs

After removing blood, livers were excised and homogenized with 0.05% collagenase IV (Sigma–Aldrich, Saint-Louis, Missouri) buffered with 0.1 M HEPES for 20min at 37°C. Hepatocytes were removed by a short centrifugation at 50g. The non-parenchymateous cells (NPCs) were filtered through a 70 μm filter and resuspended in PBS 2% Fetal Cow Serum prior to staining with monoclonal antibodies and flow cytometry analysis. For KCs enrichment, NPCs were resuspended with Optiprep 22% (Axis-Shield, Scotland), layered with HBSS/ EDTA (5mM) and centrifuged at 900g at room temperature for 20min. Among NPCs, lymphocytes CD45^+^ count was quantified by the labeling of CD45 cell surface antigen (V500-conjugated rat anti-CD45 monoclonal antibody 30-F11, Becton Dickinson, New Jersey). Macrophages were stained with a rat anti-F4/80 antibody (CI:A3-1, AbD Serotec, Kidlington, UK) and a rat anti-MHCII antibody (M5/114.15.2, Ebiosciences, San Diego, USA), a rat anti-CD40 (3_23) and anti-CD86 (GL1) monoclonal antibodies, an armenian hamster anti-CD80 (16-10A1) and anti-CD11c (HL3) mononucleal antibodies (Becton Dickinson, New Jersey, USA). Results were analyzed with a Fortessa LSR-II machine (Becton Dickinson Immunocytometry Systems, New Jersey, USA).

### Measurement of liver triglycerides and glycogen

Portions of frozen liver from mice were homogenized in chloroform-methanol (2:1) in order to extract total lipids and TGs were separated by thin layer chromatography. TGs were extracted from the silica plate with acetone, measured with a colorimetric diagnostic kit (Triglycerides FS; Diasys, Holzheim, Germany) and expressed in mg of TGs per mg of liver. To quantify liver glycogen, we used a glycogen assay kit (MAK016, Sigma-Aldrich, Missouri, USA). Briefly, 10 mg of liver is homogenized in 100 μL of water at 4°C and boiled for 5 minutes to inactivate enzymes. Samples were centrifugated at 13,000g for 5 min. 50 μL of samples were incubated in the Master mix as described by the purchaser. The absorbance was measured at 570 nm. Glycogen measurement was performed on mice after 12 weeks of diet, since livers of mice after 16 weeks of dier were used for other experiments.

### Liver histology

Liver were fixed overnight at 4°C in 4% paraformaldehyde and then embedded in paraffin. Paraffin sections (4 μm) were stained with Hematoxylin and Eosin (H&E). Images were obtained by using a NanoZoomer Digital Pathology system (x20), objective lens numerical aperture 0.75 (Hamamatsu Photonic).

### Reverse transcription of RNA

Mice livers were disrupted in Qiazol solution. Total RNA was extracted using a Qiagen RNeasy Lipid tissue minikit (Qiagen, Toronto, Canada). The RIN (RNA integrity number) was determined with the Agilent bioanalyzer 2100 system with the RNA 6000 Nano Labchip kit (Agilent Technologies, Santa Clara, Californie). Samples with a RIN inferior to 8 were eliminated. For cDNA synthesis, 1 μg of each total RNA sample was reverse transcribed. After their denaturation (5 min at 70°C), a 12 μl mix containing 1 μg of RNA, 1 μl of M-MuLv reverse transcriptase, 4 μl of 5x Buffer, 2 μl of 0.1 M dithiothreitol, and 1 μl of Protector RNase Inhibitor (40 U/μl), 10 mM dNTP Mix (Invitrogen, Carslbad, Canada) and random hexamers (Roche Diagnostics, Meylan, France), was prepared for each sample. Samples were then heated at 65°C for 5 min and cooled on ice. The reaction conditions were 42°C during 1h, 5 min at 94°C.

### Gene expression analysis by quantitative PCR

Real-time qPCR was performed in a Light Cycler 480 (Roche Diagnostics, Risch, Swiss) using the LC FastStart DNA Master SYBR Green I kit (Roche Diagnostics). Amplification was initiated with an enzyme activation step at 95°C for 10 min, followed by 40 cycles consisting of 20s of denaturation at 95°C, 15s of annealing at the temperature appropriate for each primer, and 10s of elongation at 72°C. Primers used to amplify cDNA are listed in Table 1. Data were analyzed using LC 480 Software (Roche Diagnostics). The relative genes expression were normalized to the GAPDH reference gene.

**Table 1:** Primers used for q-PCR reactions.

Name	Forward	Reverse
**GAPDH**	5'-GTG-GAC-CTC-ATG-GCC-TAC-AT-3'	5'-TGT-GAG-GGA-GAT-GCT-CAG-TG-3'
**ACC**	Ref Qiagen: QT00258419	
**FAS**	5’-TTC-CAA-GAC-GAA-AAT-GAT-GC-3’	5’-AAT-TGT-GGG-ATC-AGG-AGA-GC-3’
**CPT1**	5’-TCT-TGC-AGT-CGA-CTC-ACC-TT-3’	5’-TCC-ACA-GGA-CAC-ATA-GTC-AGG-3’
**PPAR-**γ	5'-GCA-GCT-ACT-GCA-TGT-GAT-CAA-GA-3'	5'-GTC-AGC-GGG-TGG-GAC-TTT-C-3'
**ChREBP**	5'-ATG-ACC-CCT-CAC-TCA-GGG-AAT-A-3'	5'-GAT-CCA-AGG-GTC-CAG-AGC-AG-3'
**SREBP-1c**	5’-AAC-GTC-ACT-TCC-AGC-TAG-AC-3’	5’-CCA-CTA-AGG-TGC-CTA-CAG-AGC-3’
**TNF**α	5’-TCT-CAT-CAG-TTC-TAT-GGC-CC -3’	5’-GGG AGT-AGA-CAA-GGT-ACA-AC-3’
**IL6**	5’-GTT-CTC-TGG-GAA-ATC-GTG-GA-3’	5’-TGT-ACT-CCA-GGT-AGC-TAT-GG-3’
**F4/80**	5'-TCT-GGG-GAG-CTT-ACG-ATG-GA-3'	5'-GAA-TCC-CGC-AAT-GAT-GGC-AC-3'
**CD68**	5’-CTT-CCC-ACA-GGC-AGC-ACA-G-3’	5’-AAT-GAT-GAG-AGG-CAG-CAA-GAG-G-3’
**CCL2**	5’ AGG-TCC-CTG-TCA-TGC-TTC-TG-3’	5’-TCT-GGA-CCC-ATT-CCT-TCT-TG-3’
**CCR2**	Ref Qiagen: QT02276813	
**Nos2**	5’-CCA-AGC-CCT-CAC-CTA-CTT-CC-3’	5’-CTC-TGA-GGG-CTG-ACA-CAA-GG-3’
**Arg1**	5’-CTC-CAA-GCC-AAA-GTC-CTT-AGA-G 3’	5’-AGG-AGC-TGT-CAT-TAG-GGA-CAT-C-3’
**MRC1**	5'-GGA-CGA-GCA-GGT-GCA-GTT-3'	5'-CAA-CAC-ATC-CCG-CCT-TTC-3'

### Feces collection and analysis of the gut microbiota

Fresh fecal samples from mice fed the different diets were collected and fixed one day before mice were euthanized. Fluorescent in Situ Hybridization combined with Flow Cytometry (FISH-FCM) analysis was performed. Fixation and permeabilization of samples were carried out as described previously [7]. Permeabilized cells were hybridized in the dark for 16 h at 37°C in 50 μl of hybridization buffer (900 mM NaCl, 20 mM Tris–HCl, pH 7.2; 0.01% SDS; 15% formamide; and 4 ng μl−1 of fluorescent probe) in a 96-well microtiter plate. The 16S rRNA-targeted oligonucleotide probes targeting nine bacterial groups used in this study are listed in Table 2, with 4 control probes, Eub338 which is specific for the domain Bacteria and NonEub338 which is the complement of Eub338 and is used as a negative control to account for non-specific binding and background fluorescence. They were covalently linked with fluorescein isothiocyanate (FITC) or indodicarbocyanin (Cy5) at the 5′-end (Thermo Fischer Scientific GmbH, Ulm, Germany). All the following steps were performed in the dark, a volume of 150 μl of hybridization solution was added to each well and cells were pelleted at 4000g for 15 min. Non-specific binding of the probe was removed by incubating the bacterial cell suspension at 37°C for 20 min in a washing solution (64 mM NaCl, 20 mM Tris–HCl, pH 8.0, 5 mM EDTA, 0.01% SDS). Cells were pelleted and resuspended in phosphate-buffered saline (PBS, pH 7.2). Aliquots of 100 μl were added to 0.5 ml of FACS Flow (Becton Dickinson, Franklin Lakes, New Jersey) for data acquisition by flow cytometry. Cell enumeration was performed by combining one group Cy5-probe with the Eub338 FITC-probe in one hybridization tube. Results were expressed as cells hybridizing with group-Cy5 probe as a proportion of the total bacteria hybridizing with the general Eub338 FITC-probe. To quantify bacterial DNA, optimized buffers and enzymes were used to digest the liver and to recover DNA. Bacterial DNA was quantified by using universal primers for Eubacteria: ACT-CCT-ACG-GGA-GGC-AGC-AGT 3' (forward) and 5' ATT-ACC-GCG-GCT-GCT-GGC 3 (reverse).

**Table 2:** Nomenclature, sequences and targets of probes used for fluorescent in situ hybridization.

*Probe name*	*Sequence (5’-3’)*	*Target*	*Fluorochrome*
NON EUB 338	ACATCCTACGGGAGGC	none	5’FITC
NON EUB 338	ACATCCTACGGGAGGC	none	5’CY5
EUB 338	GCTGCCTCCCGTAGGAGT	domain Bacteria	5’FITC
EUB 338	GCTGCCTCCCGTAGGAGT	domain Bacteria	5’CY5
Bif164	CATCCGGCATTACCACCC	Bifidobacterium genus	5’CY5
Bac303	CCAATGTGGGGGACCTT	Bacteroides-Prevotella group	5’CY5
Lab158	GGTATTAGCAYCTGTTTCCA (*Y = C/T)	Lactobacillus-Lactococcus-Enterococcus group	5’CY5
Clep 866	GGT GGA TWA CTT ATT GTG Competitor 1: GGT GGA AWA CTT ATT GTG Competitor 2: GGT GGA TWAV CTT ATT GCG (*W = A/T)	Clostridium leptum subgroup	5’CY5
Ato 291	GGTCGGTCTCTCAACCC	Atopobium cluster and Coriobacterium group	5’CY5
Enter 1432	CTTTTGCAACCCACT	Enterobacteria	5’CY5
Erec 482	GCTTCTTAGTCAGGTACCG	Clostridium coccoides group	5’CY5
Ecyl 387	CGCGGCATTGCTCGTTCA	Erysipelotrichi	5’CY5
MIB 661	GCATTCCGCATACTTCTC	Mouse Intestinal Bacteria	5’CY5

### Statistical analysis

Results are represented as mean±SEM. Statistical analysis were performed by Mann-Whitney and Kruskal-Wallis tests (Graphpad Prism, Graphpad Software Inc, La Jolla, California); p<0,05 was considered statistically significant. The NCSS9 software (Kaysville, Utah) was used for principal component analysis (PCA).

## Results

### Fructose involvement in body weight and glucose sensitivity

**Fig 1:**
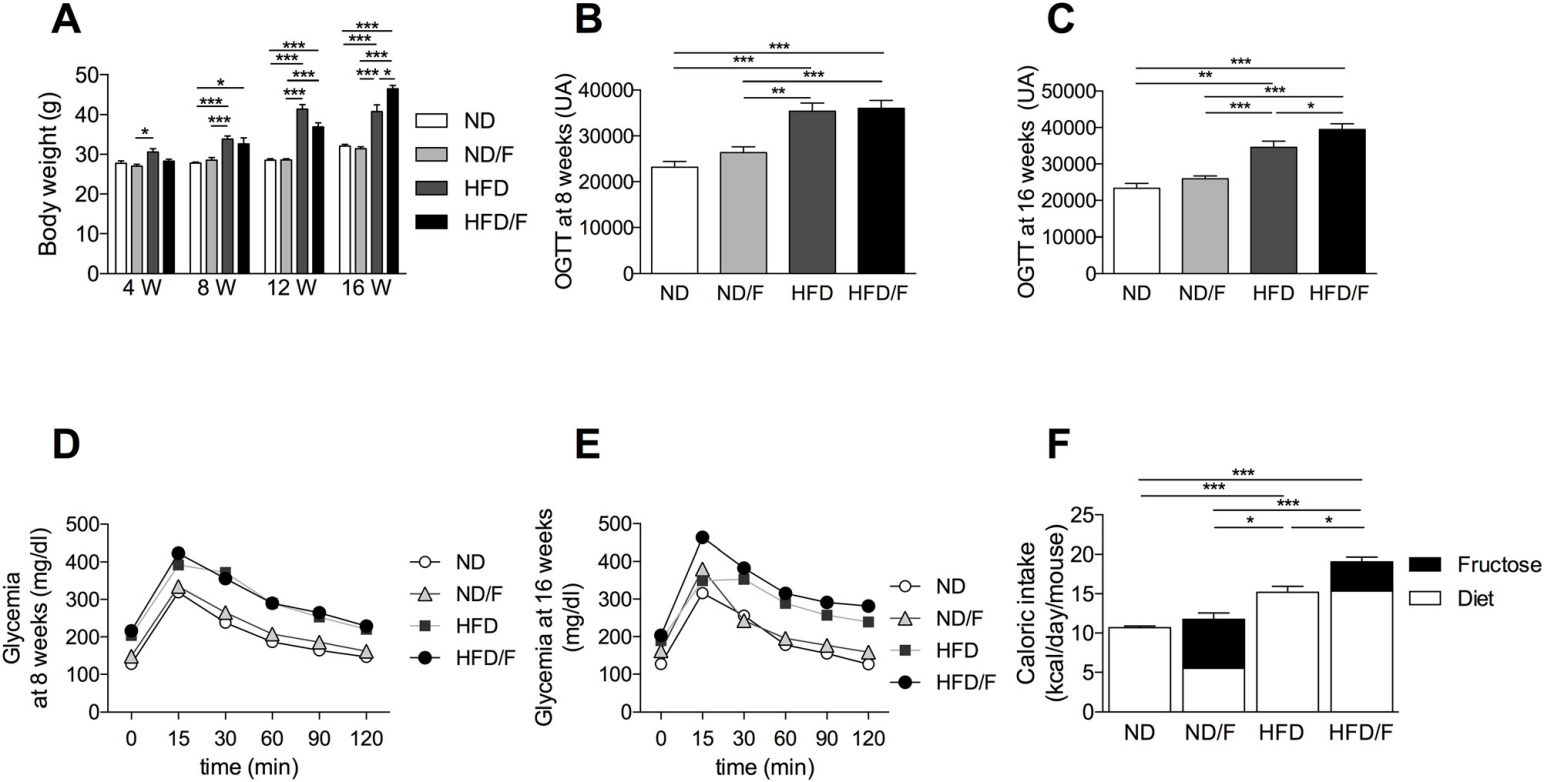
Follow-up of mice after 4, 8, 12 and 16 weeks period of feeding with ND, ND/F, HFD and HFD/F diets. Mice were fed with normal diet (ND), ND with fructose (ND/F), high fat diet (HFD) and HFD with fructose (HFD/F) diets during 4, 8, 12 and 16 weeks. **(A)** Evolution of mice weight. **(B)** Representative histograms of the area under the curve of the oral glucose tolerance test after 8 weeks of diet or **(D)** at 16 weeks of diet. (**C**) Curves of glycemia after an oral glucose tolerance test at 8 weeks of diet or (**E**) at 16 weeks of diet. **(F)** Caloric intake. Data represent the mean±SEM of 8 mice, *: p<0.05, **: p<0.01, ***p < 0.001.

After 8 weeks of diet, high fat diet (HFD) fed mice showed a significant increase in body weight compared to mice fed with a normal diet (ND) or a ND diet associated with fructose (ND/F) (Fig 1A). This body weight increase was associated with an increase of the glucose sensitivity in mice fed with a ND diet compared to mice fed with a HDF diet, independently of fructose (Fig 1B and 1C). After 12 weeks of diet, the body weight increased in the HFD and the HFD/F compared to ND and ND/F fed mice groups (Fig 1A). After 16 weeks of diet, the body weight of mice were not modified in the ND/F fed mice group compared to the ND fed mice group and the caloric intake was similar in the 2 groups (Fig 1A and 1F). Altough the fructose participated in a large part of the calories number/day in the ND/F fed mice group (53%), the fructose consumption did not modify the glucose sensitivity in mice (Fig 1D and 1E). HFD/F fed mice group showed a significant weight gain compared to the HFD fed mice group. This difference was associated with a significant increase of the caloric intake of mice fed with the HFD compared to the HFD/F diet (Fig 1F). Similarly, the glucose sensitivity of the group of mice fed with the HFD/F was significantly higher compared to the HFD fed mice group (Fig 1D and 1E). The fructose consumption in the HFD/F fed mice group participated in a low part of the calories number/day compared to the ND/F fed mice group (19,5% and 53% of the calories intake, respectively) but induced a higher caloric intake and a worsening of the glucose sensitivity (Fig 1F).

### Liver lesions associated with diet composition

**Fig 2:**
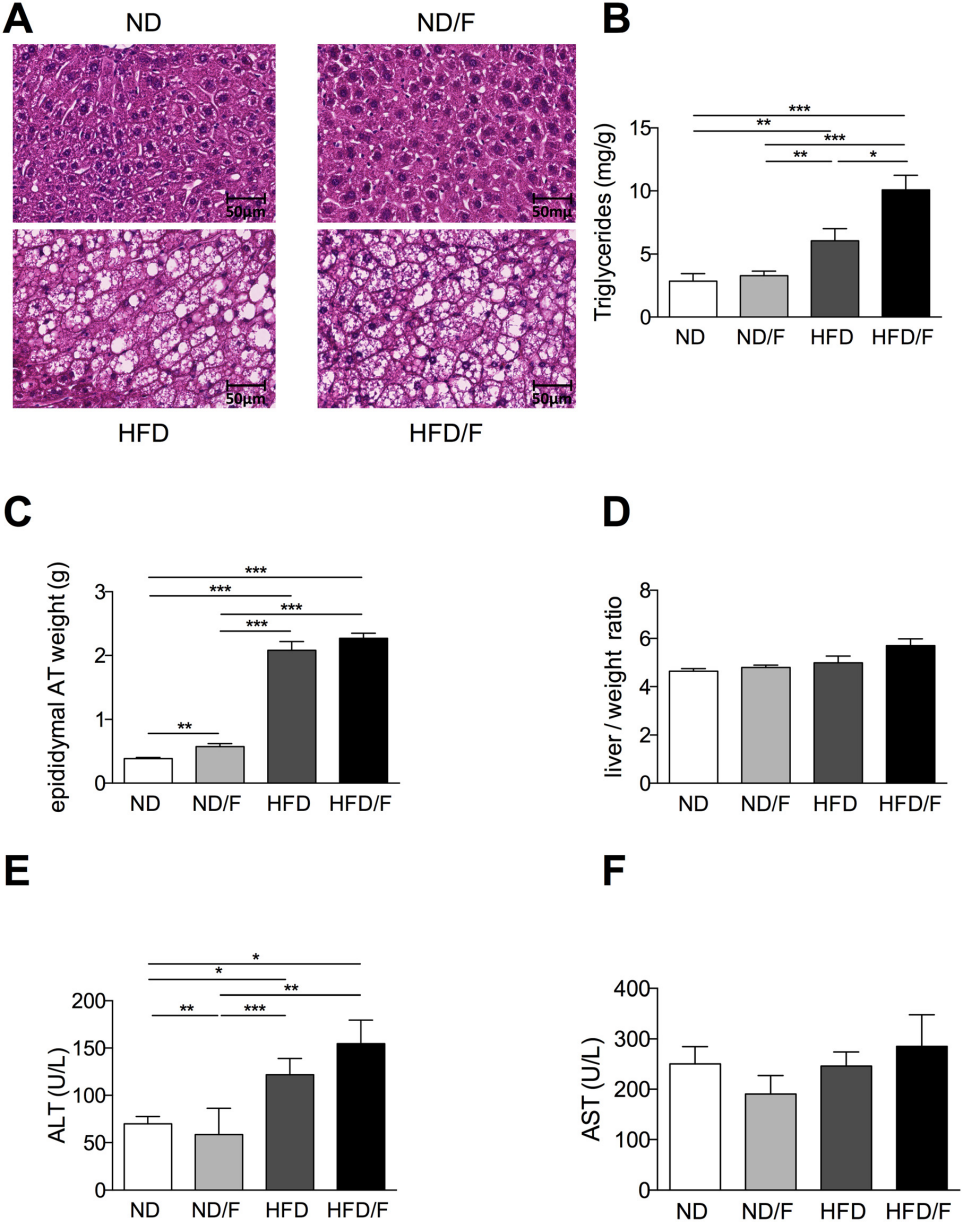
Analysis of hepatic phenotype and fat mass after 16 weeks of diet. **(A)** Histological examination of liver tissue section stained with Hematoxylin-eosin (scale 50um). **(B)** Hepatic triglyceride content. **(C)** Fat mass of epididymal adipose tissue amount. **(D)** Liver / weight ratio. **(E)** Plasma ALT level. **(F)** Plasma AST level. Normal diet: ND; ND with fructose: ND/F; high fat diet: HFD; and HFD with fructose: HFD/F. Data represent the mean±SEM of 8 mice, *: p<0.05, **: p<0.01, ***p < 0.001.

**Fig 3:**
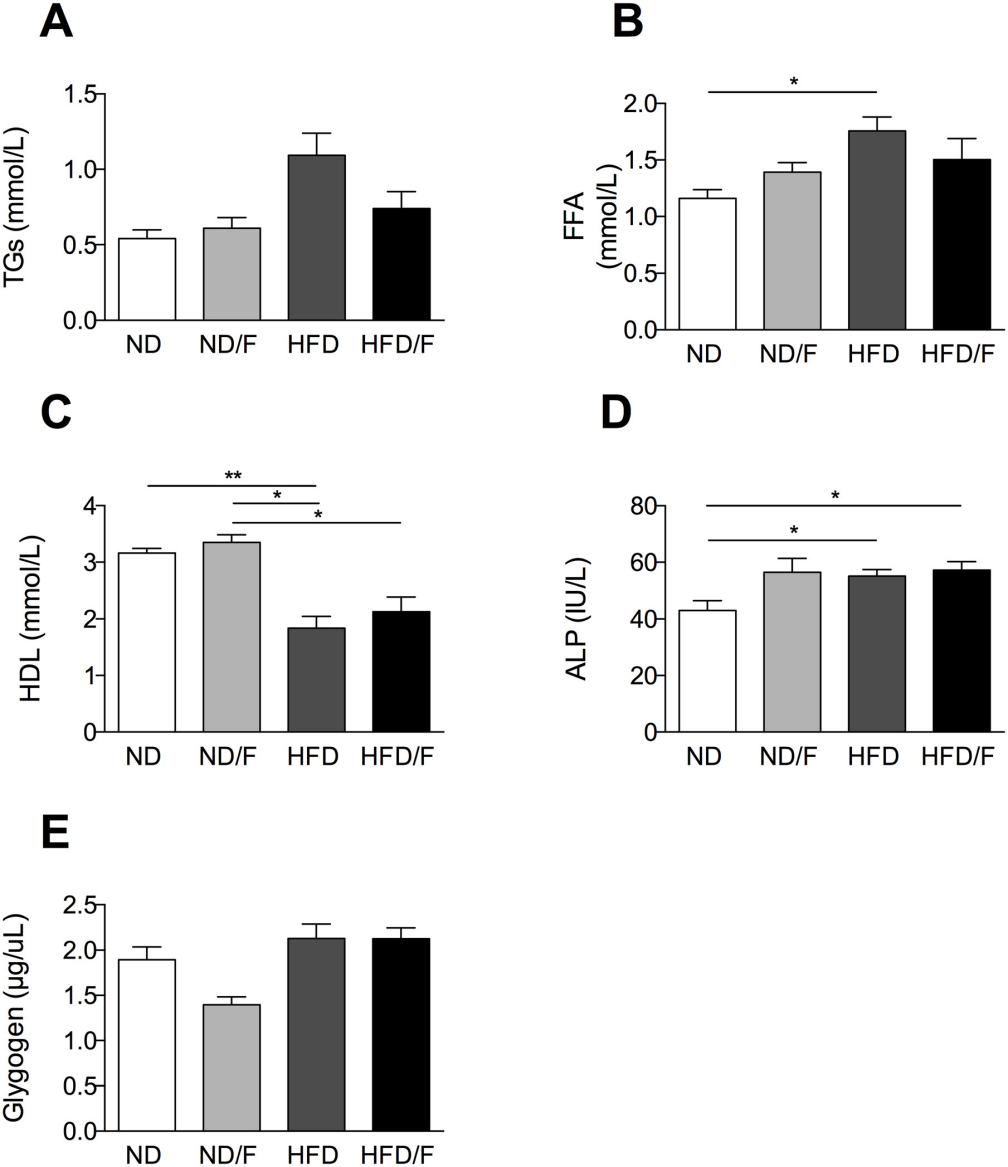
Quantification of plasma parameters and liver glycogen. Several parameters were quantified in plasma **(A)** Triglycerides, **(B)** Free fatty acid, **(C)** HDL, **(D)** Alkaline phosphatase (ALP)**. (E)** Liver glycogen in mice after 12 weeks of diet. Normal diet: ND; ND with fructose: ND/F; high fat diet: HFD; and HFD with fructose: HFD/F. Data represent the mean±SEM of 8 mice (Mann-Whitney, *: p<0.05, **: p<0.01).

After 16 weeks of diet, mice were euthanized and liver lesions were assessed. Steatosis as shown by histology and TGs quantification was significantly higher in the HFD/F group of mice (Fig 2A and 2B) compared to the other groups of mice. Conversely, fructose associated with the ND diet was not able to induce a higher steatosis compared to the ND group of mice (Fig 2B). However, if body weight gain was similar between the ND and the ND/F groups of mice, the epidydimal adipose tissue (AT) weight was slightly increased in the ND/F group of mice (Fig 2C). Conversely, the epididymal AT was similar in the HFD and the HFD/F group of mice although a high caloric intake in the HFD/F group of mice. Hepatocellular injury induced an increase of ALT only in both groups of HFD fed mice independently of the fructose consumption compared to the ND fed mice groups (Fig 2E), AST level was not modified (Fig 2F). The liver body / weight ratio were not modified by diets as often described in diet induced obesity in mice conversely to *ob/ob* mice or diet deficient in choline and methionine (Fig 2D) [8, 9]. Plasma triglycerides were increased exclusively in the HFD fed mice, as the free fatty acids (Fig 3A and 3B), conversely to the liver triglycerides which were higher in the HFD/F fed mice (Fig 2B). HDL was decreased in both HFD group of mice (Fig 3C). Alkaline phosphatase was increased in all groups of mice compared to the control group (Fig 3D). As fructose could modify glycogen storage we quantified the liver glycogen. Surprisingly, the level of glycogen was not modified in mice whatever the diet (Fig 3E).

**Fig 4:**
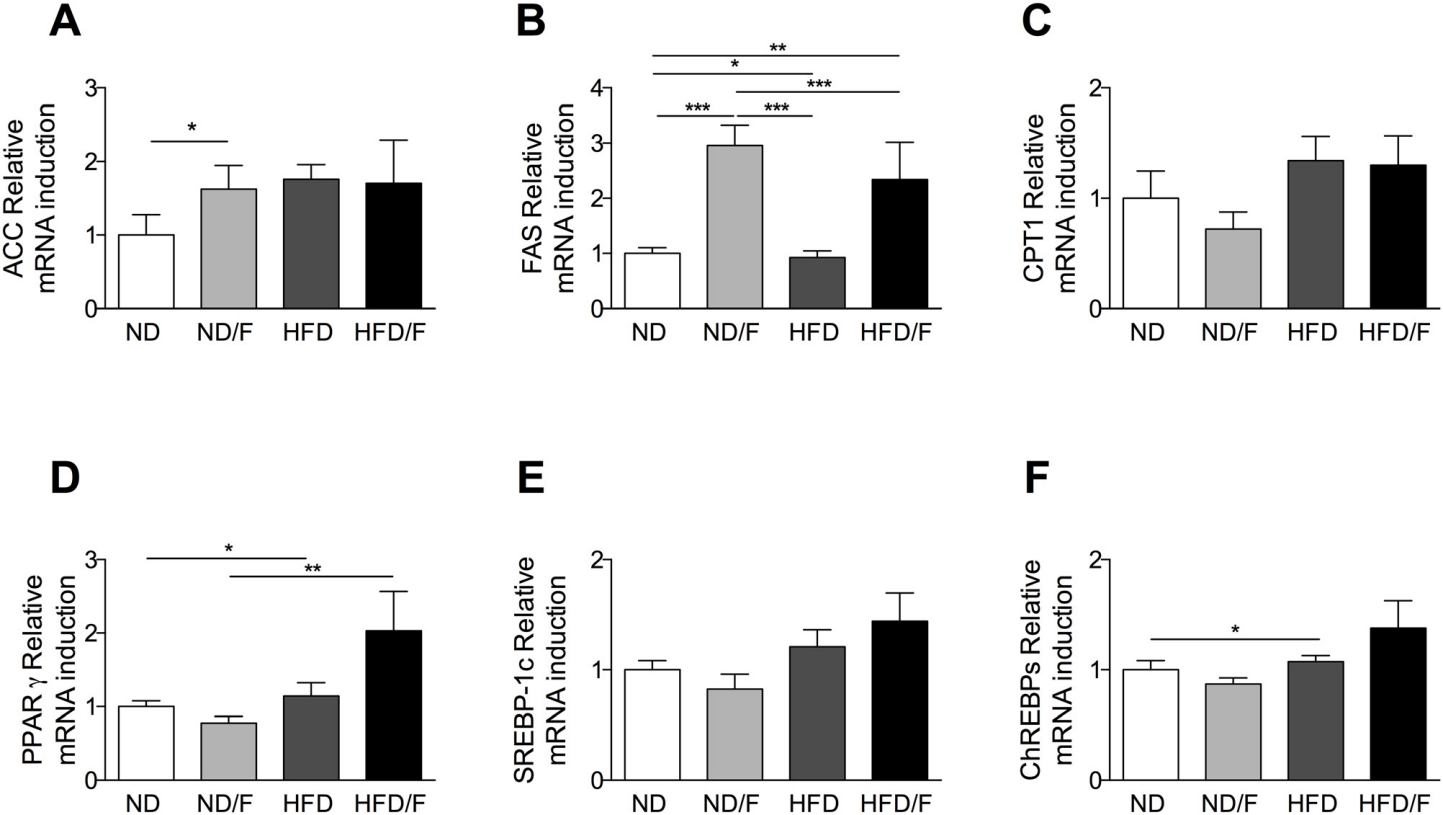
Quantification of liver mRNA of genes involved in lipogenesis. **(A)** Acetyl-CoA-carboxylase (ACC), **(B)** Fatty acid synthase (FAS), **(C)** Carnitine palmitoyl transferase 1 (CPT1), **(D)** Peroxysome proliferator activated receptor gamma (PPARγ), **(E)** Sterol regulatory element binding protein 1c (SREBP-1c) and **(F)** Carbohydrate-responsive element-binding protein. Normal diet: ND; ND with fructose: ND/F; high fat diet: HFD; and HFD with fructose HFD/F. Data represent the mean±SEM of 8 mice (Mann-Whitney, *: p<0.05, **: p<0.01, ***p < 0.001).

We assessed to the *de novo* lipogenesis by mRNA quantification of ACC (Acetyl-CoA-carboxylase) and FAS (Fatty acid synthase) enzymes. FAS showed a significant increase in mice fed with fructose and a tendency in mice fed with a HFD and a HFD/F diets (Fig 4A and 4B). However, mice fed with a HFD diet did not show a similar increase of FAS. We found an increase of lipogenesis in mice fed with fructose without an increase of TG in the liver. No modification of the CPT1 was observed (Fig 4C). The quantification of mRNA expression for nuclear factors involved in lipogenesis regulation showed that PPARγ was specifically increased in mice fed with a HFD and a HFD/F diets (Fig 4D). No increase was observed for SREBP1c and a slight increase was found for ChREBPs in mice fed with a HFD diet (Fig 4E and 4F). These discrepancies were previously described in several papers [4, 10–12].

**Fig 5:**
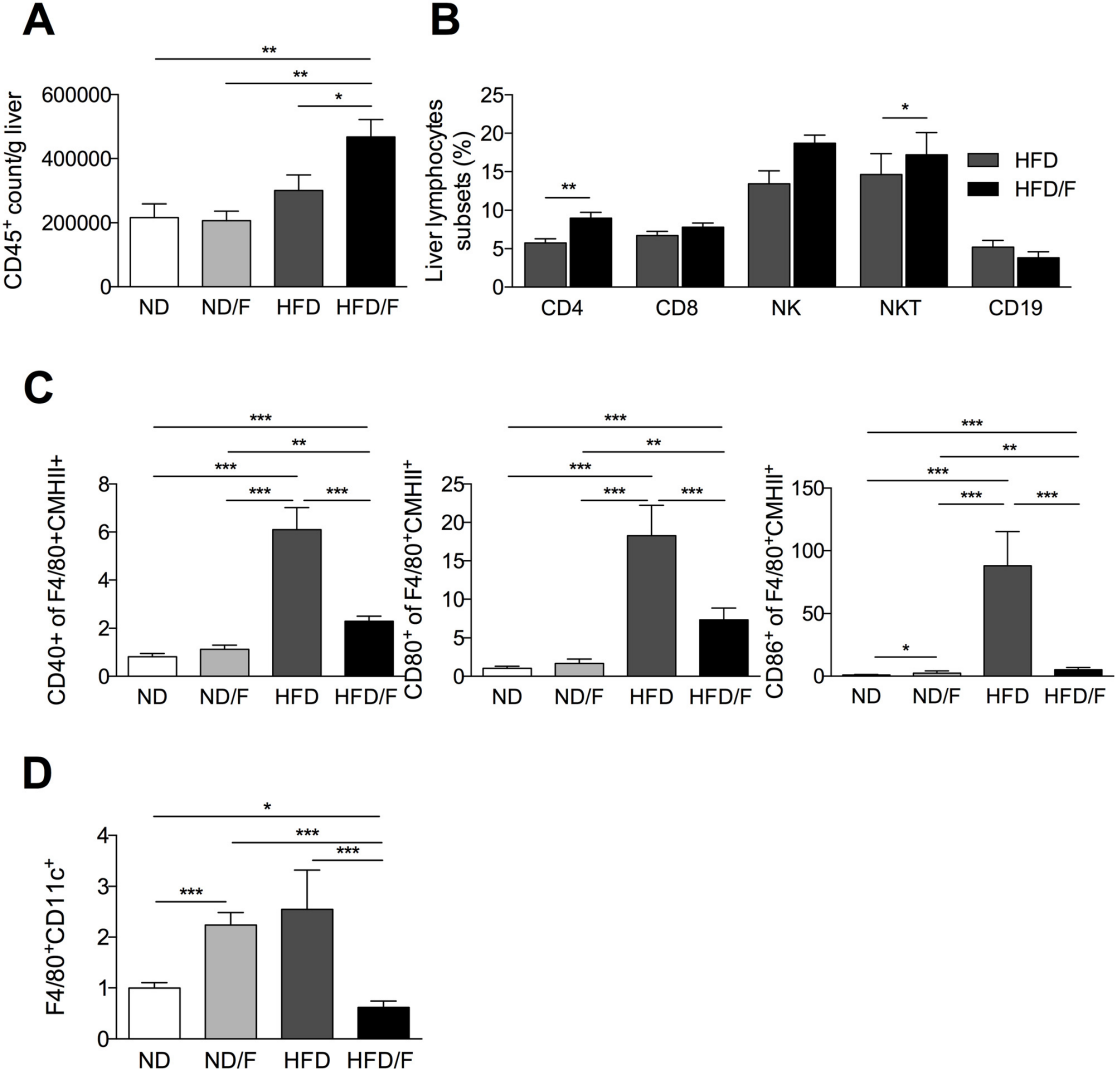
Quantification of inflammation and KC phenotype after 16 weeks of diet. Quantification of liver lymphocytes subsets in the liver of mice by flow cytometry, **(A)** CD45^+^, **(B)** CD4^+^, CD8^+^, CD19^+^, NKT and NK cells. **(C)** Quantification of activated KCs phenotype by analysis the up-regulation of co-stimulatory molecules CD40, CD80, CD86 and CMHII by flow cytometry after 16 weeks on their respective diets. **(D)** Percentage of KCs expressing CD11c. Normal diet: ND; ND with fructose: ND/F; high fat diet: HFD; and HFD with fructose: HFD/F. Data represent the mean±SEM of 8 mice (Mann-Whitney, *: p<0.05, **: p<0.01, ***p < 0.001).

**Fig 6:**
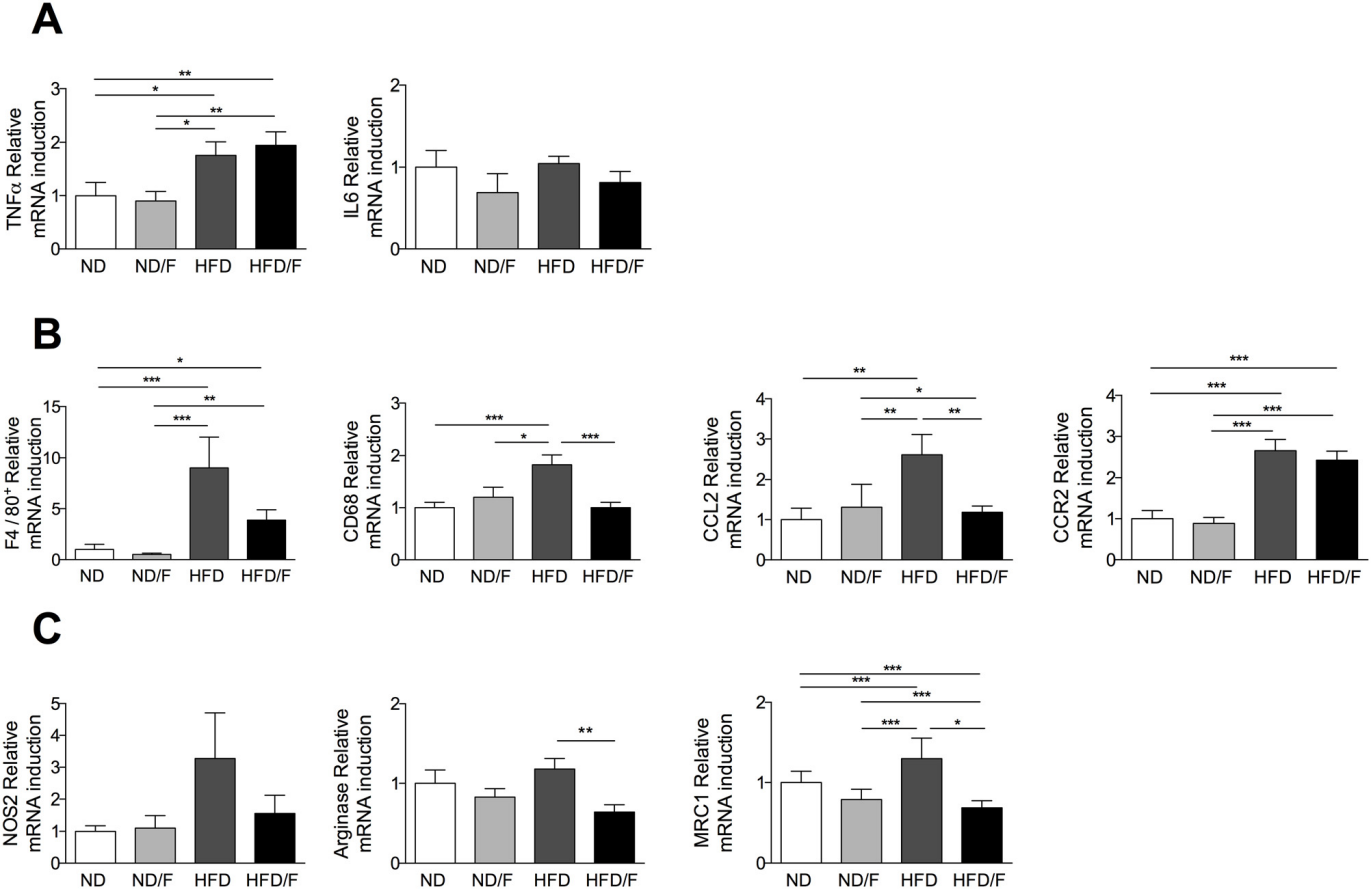
Quantification of inflammation and KC phenotype after 16 weeks of diet. Hepatic mRNA level of genes involved in inflammation and in KC phenotype. mRNA levels were normalized to GAPDH expression and fold inductions were calculated. **(A)** TNFα, IL6. **(B)** F4/80, CD68, CCL2 and CCR2. **(C)** NOS2, Arginase and MRC1. Normal diet: ND; ND with fructose: ND/F; high fat diet: HFD; and HFD with fructose: HFD/F. Data represent the mean±SEM of 8 mice (Mann-Whitney, *: p<0.05, **: p<0.01, ***p < 0.001).

As ALT level is not strictly correlated with the severity of liver injury in NAFLD [4, 8], inflammation was also scored by quantification of intrahepatic lymphocytes. Fructose consumption associated with a HFD diet (HFD/F) induced a higher lymphocyte recruitement compared to a HFD diet (Fig 5A). Lymphocytes increased was characterized by higher levels of lymphocytes T CD4+ and NKT (Fig 5B). This inflammation was associated with the increase of mRNA expression of TNFα in the two HFD group of mice (Fig 6A) although IL6 mRNA expression was not different as previously shown in HFD mice [4]. However, the F4/80, CD68 and CCL2 mRNA expression were significantly increase in the HFD fed group of mice compared to the HFD/F fed mice suggesting that KCs were differently activated (Fig 6B). CCR2 mRNA was increased similarly in HFD and HFD/F fed mice. As we previously demonstrated that KCs showed a proinflammatory phenotype in the liver of obese mice [4], we assessed their activated phenotype by flow cytometry. Conversely to the lymphocytes quantification, the up-regulation of co-activation molecules, CD40, CD86 or MHCII was significantly increased in KCs (F4/80 positive cells) of the HFD fed mice group (Fig 5C). The mRNA expression of genes associated with a pro (NOS2) or an anti-inflammatory phenotype (Arginase and MRC1) were modified only in KCs of HFD fed mice (Fig 6C). Moreover, as described in the white adipose tissue of obese mice, the percentage of KCs expressing the CD11c molecule was higher in the HFD fed group of mice but not in the HFD/F fed group of mice [13]. The number of CD11c KCs was also surprisingly higher in the ND/F fed group of mice without any changes in their phenotype profiles assessed by flow cytometry (Fig 5C).

### A dysbiosis was associated with fructose consumption

**Fig 7:**
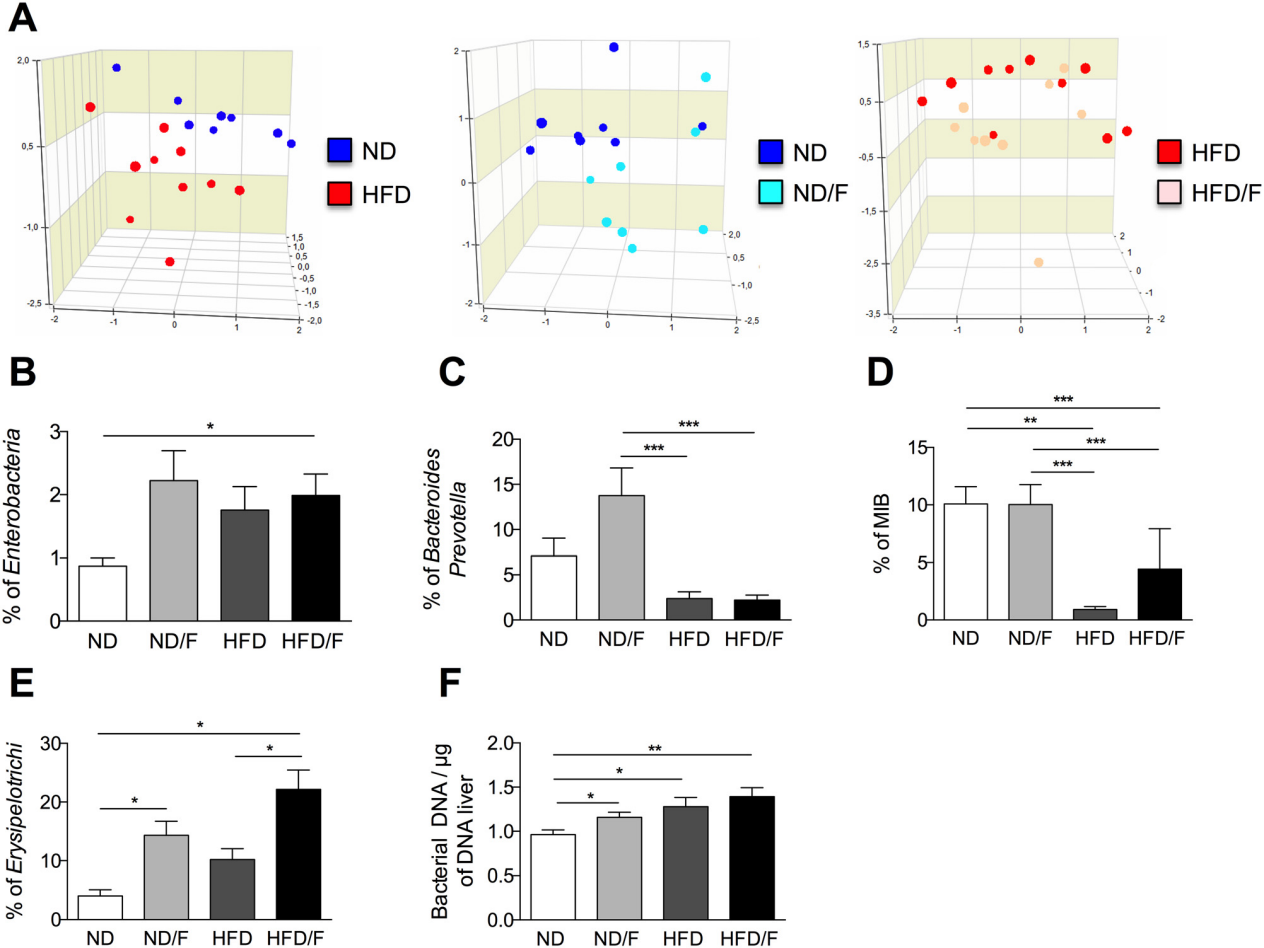
Dysbiosis induced by HFD and fructose diets after 16 weeks of diet. Analysis of gut bacterial communities by 16S rDNA analysis in feces from mice fed with different diets. **(A)** PCA representation of microbiota profile. Each point corresponds to a community from a single mouse fed with different diet. **(B)** Percentages of *Enterobacteria*. **(C)** Percentages of *Bacteroides/Prevotella* **(D)** Percentages of Mouse intestinal Bacteria. **(E)** Percentages of *Erysipelotrichi*. **(F)** Quantification of liver bacterial DNA. Data represent the mean±SEM of 8 animals (Mann-Whitney, *: p<0.05, **: p<0.01, ***p < 0.001).

As recently shown, KCs participate in the commensal clearance of blood commensal bacteria as known as the microbiota [14]. To assess that the activation of KCs could be correlated to a specific microbiota we performed FISH-FCM to analyze dominant and sub-dominant bacterial populations. A set of 12 probes targeting bacterial 16S rRNA were used to assess the relative proportion of intestinal bacterial phylogenetic groups (Table 2). The composition of the faecal microbiota were similar in all groups of mice before the diet (data not shown). Principal component analysis (PCA) was used to examine the overall structure of the microbiota. Resulting ordination plots highlighted that dysbiosis were associated with the diet composition (Fig 7A). However, dysbiosis was clearly observed by addition of fructose to a ND diet or the addition of lipids to a ND diet. Indeed, the HFD consumption compared to the ND consumption was associated with a dysbiosis which clustered each group of mice ND versus HFD groups in accordance to previous data [15, 16]. Similarly, the addition of fructose to a ND diet clustered mice in two separate groups. However, the addition of fructose to the HFD diet (HFD/F) showed that diversity of nutrients (high lipids and high carbohydrates) decrease the ability to cluster groups of mice by their gut microbiota (Fig 7A). Among analysed bacteria, the group of *Clostridium coccoides*, *Clostridium leptum*, *Atopobium*, *Lactobacillus-Enterococcus* and *Bifidobacterium* were not significantly modified by diets (data not shown). Conversely, *Enterobacteria* were systematically increased by changes in the diet (Fig 7B). As previously shown *Bacteroides* genus was decreased by the HFD diet independently of fructose consumption (Fig 7C). Conversely, the consumption of fructose with a normal diet induced a high increase of *Bacteroides*. *Erysipelotrichi* were increased by the fructose consumption independantly of the HFD diet (Fig 7D). Mouse Intestinal Bacteria (MIB) which were also detected in humans, were decreased in HFD and HFD/F fed group of mice (Fig 7E). However, the fructose consumption with the HFD prevented the large decrease of the MIB percentage. To assess the disruption of the gut barrier, we quantified the bacterial DNA in the liver (Fig 7F). We showed that bacterial DNA content of mice fed a HFD was significantly different from the DNA content of mice fed a ND. However, the DNA content was also increased in mice fed with the ND diet and fructose (ND/F). Although this is an indirect quantification of the gut barrier disruption, this result prove that the increase of bacterial DNA translocation in HFD mice could explain the higher activation of KCs. Then, the development of liver lesions was associated with an increase of bacterial DNA content but also with specific bacterial patterns.

## Discussion

Nutrients have an influence on the body and recently changes in food consumption induced the development of various pathologies including obesity-induced liver disease. To counteract the increasing high fat consumption and the excess of calories intake, industrialized food was designed to compensate high energy content by lowering sugar consumption. If non caloric artificial sweeteners were used for many years, the use of fructose as alternative sugar source was more recent. Recent studies showed that fructose promote increased food intake and contributed to impair insulin sensitivity [17] and was associated with the severity of NAFLD [18]. However, the effects of fructose and the higher energy intake could be confounded especially in humans studies [19]. To address the impact of fructose associated with a high fat diet, we induced obesity in mice and also adressed the impact of fructose alone. Fructose did not induce an increase of the amount of caloric intake per day. Actually, in mice fed a ND/F diet compared to mice fed a ND diet, food consumption was decreased whereas water consumption containing 20% of fructose was increased, leading to an absence of body weight gain. This fructose consumption was not sufficient to impair the glucose tolerance as previously described [11, 20]. Conversely, mice fed a HFD with fructose showed an increase of ingested calories per day. In the HFD and HFD/F group of mice, steatosis was significantly increased. Expression of some genes involved in lipid homeostasy, *de novo* lipogenesis and transcriptional factors showed that FAS and PPARγ were increased specifically in mice fed a HFD/F diet. Surprisingly, we found that *de novo* lipogenesis was increased in mice fed fructose whereas no TGs accumulation was found. Moreover, we did not found any significant increase of glycogen storage in mice fed with fructose after 12 weeks of diet. The increase of steatosis in both group of mice fed with a HFD diet was associated with differences in the onset of inflammation. Actually, we showed that the lymphocyte infiltration induced by the HFD/F diet was increased compared to the HFD fed mice group. Conversely, and surprisingly Kupffer cells which were activated in the HFD fed mice group were not activated in the HFD/F group. We have previously shown that an HFD diet induced not only lymphocytes recruitement but also KCs modifications [4]. KCs as monocytes/macrophages are a heterogenous population which release a high diversity of cytokines and chemokines, express numerous cell surface markers and transcriptional profiles. These cells have been classified either into pro-inflammatory, M1 or anti-inflammatory, M2 macrophages. However, this simple dichotomous nomenclature does not reflect the diversity of macrophages in a tissue [21–23]. KCs from HFD mice accumulated lipids which were responsible for their inflammatory phenotype. In the present study we showed that fructose differently induced the KCs phenotype. Transcriptional profile was clearly modified in KCs of HFD fed mice compared to mice fed a HFD and fructose diet. As an antigen presenting cell, KCs could process antigens inducing a higher expression of surface co-stimulatory molecules. The analysis by flow cytometry of co-stimulatory molecules CD80, CD86 and CD40, also showed a specific increase in HFD fed mice. Indeed, HFD/F diet induced a higher TG accumulation in the liver, that was associated with a lower activation of KCs compared to mice fed a HFD diet. However, we observed a higher content of lymphocytes in the liver of those mice and especially high percentages of CD4^+^ lymphocytes and NKT cells. NKT cells are known for their potent role in immune regulation, especially in the liver where the NKT content is high in comparison to the blood NKT content. In liver disease, a decrease in NKT percentage was described in *ob/ob* mice and conversely increased in alcohol-induced liver disease [24, 25]. In *ob/ob* mice the low content of NKT in liver was associated with an excessive production of pro-inflammatory cytokines promoting inflammation [24]. Mice fed a HFD/F diet showed a lower activation of KCs although metabolic profile, TG content and glucose sensitivity were similarly promoted compared to HFD fed mice. We did not assessed the phenotype of NKT cells in HFD/F fed mice, but their increase number could be associated with a pro-inflammatory phenotype dampering KC activation.

Then, nutrients were able to differently induce liver inflammation pathways. We found that these discrepancies were associated with specific changes in the composition of the gut microbiota. It has been described that *Bacteroidetes* were decreased in obese and associated with liver inflammation [16, 26, 27]. However, a more recent study in obese adolescent showed a significant increase of *Bacteroidetes* [28]. As fructans, which are polymers of fructose, favored the *Bacteroides* growth [29], the amount of fructose in the daily intake could explained these discrepancies. The fructose diet induced specifically an increase of *Erysipelotrichi* and prevented the large decreased of MIB induced by a HFD diet. Altogether these data showed that fructose modified gut microbiota according to the fat content in the diet. Previous studies using water containing 30% fructose described a steatosis in fructose fed mice and antibiotics wera able to decrease the TG accumulation [10]. They also showed a dependance on Toll like receptor 4 (TLR4) expression suggesting a possible role of microbiota [30]. Then, as the dysbiosis was different between mice consuming fructose or not, we suggest that bacteria and microbial patterns could act differently to activate KCs as we shown here. As it has been shown that DNA bacterial content was increased in HFD fed mice, we assessed the bacterial DNA in livers and confirmed that bacterial DNA content was increased [31]. Fructose did not induced significant differences in DNA translocation reinforcing the importance of bacteria species in deleterious effect of microbiota and their role on KCs activation. Indeed, KCs by their phagocytic function clear bacterial compounds from the systemic vasculature. HFD diet induced a dysregulation of lipid metabolism associated with a pro-inflammatory phenotype and disturbances of phagocytic functions [4, 32]. Then, the clearance of bacterial patterns, and more specifically patterns of specific bacterias, was modified and could participate in liver damage [14].

In conclusion, we showed that fructose induced differential dysbiosis in HFD fed mice which seemed lowering activation of KCs but inducing a direct higher recruitement of intrahepatic lymphocytes. As the microbiota composition is modified by the diet composition, identification of microbial patterns and metabolites produced by the gut microbiota appears to be a new challenge to decipher mechanisms involved in inflammatory liver damage rather than the only identification of specific bacterial species. Specific patterns will probably assigned in liver disease and will clarify the role of KCs in dampering the liver damage or not.

## Abbreviations

NAFLD: non-alcoholic fatty liver disease
NASH: non-alcoholic steatohepatitis
NKT: natural killer T cell
KCs: kupffer cells
ND: normal diet
HFD: high fat diet
OGTT: oral glucose tolerance test
TG: triglycerides
ALT: alanine aminotransferase
AST: aspartate aminotransferase
FFA: free fatty acids
HDL: high density lipoprotein
ALP: alcaline phosphatase
NPCs: non parenchymateous cells
CD: cluster of differenciation
GAPDH: glyceraldehyde-3-phosphate dehydrogenase
FISH-FCM: fluorescent in situ hybridization combined with flow cytometry
PCA: principal component analysis
ND/F: normal diet with fructose
HFD/F: high fat diet with fructose
AT: adipose tissue
ACC: acetyl-CoA-carboxylase
FAS: fatty acid synthase
PPARγ: peroxysome proliferator activated receptor γ
SREBP-1c: sterol regulatory element binding protein 1c
ChreBPs: carbohydrate-responsive element-binding proteins
TNFα: tumor necrosis factor-α
IL6: interkeukin 6
CCL2: C-C motif chemokine ligand 2
CCR2: C-C motif chemokine receptor 2
MHC: major histocompatibility complex
NOS: nitric oxid synthase
Arg: arginase
MRC1: mannose receptor 1
CPT1: carnitine palmitoyl transferase 1
TLR4: Toll-like receptor 4
